# Usefulness of magnetic resonance imaging for acute abdominal pain in a pregnant woman: A case of idiopathic renal hemorrhage

**DOI:** 10.1016/j.radcr.2023.04.011

**Published:** 2023-05-06

**Authors:** Hanami Tanabe, Taku Harada, Mori Nakai

**Affiliations:** a Center of Postgraduate Clinical Training, Showa University Koto Toyosu Hospital, Tokyo, Japan; b Department of General Medicine, Nerima Hikarigaoka Hospital, 2-5-1 Hikarigaoka Nerima-ku, Tokyo 179-0072, Japan; c Department of Diagnostic and Generalist Medicine, Dokkyo Medical University Hospital, Tochigi, Japan

**Keywords:** Acute abdominal pain, MRI, Pregnancy, Ureteral stone, Hydronephrosis

## Abstract

Acute abdominal pain in pregnant women may complicate the diagnostic process of acute abdominal pain because of anatomical and physiological changes and limitations of computed tomography examinations related to radiation exposure. Here, we present the case of a 35-year-old female in her 10th week of pregnancy who was seen in the emergency department with unilateral abdominal pain and gross hematuria. Ultrasound detected only hydronephrosis and failed to identify ureteral stones, but magnetic resonance imaging revealed a diagnosis of idiopathic renal hemorrhage and intraductal ureteral hematoma, not ureteral stones. Although magnetic resonance imaging for pregnant women has the disadvantages of prolonged scan time and difficulty in image interpretation, no harm or complications to the mother or fetus have been reported. Magnetic resonance imaging may be considered in assessing acute abdominal pain in pregnant women, especially when the diagnosis is uncertain, based on shared decision-making with the patient and assessing the clinical situation and availability.

## Introduction

The approach to investigating acute abdominal pain during pregnancy is essentially the same as that used for the non-pregnant approach. Although acute abdominal and pelvic pain may be more common during pregnancy, several challenges to diagnosis arise because of pregnancy, such as pregnancy-related anatomical and physiological changes, obstetric complications, and fetus-related conditions [1,2]. As delayed diagnosis and treatment of acute abdominal pain in pregnant women increases maternal, fetal, and neonatal morbidity and mortality, appropriate imaging and intervention are needed [1].

Ureteral stones are the most common non-gynecologic cause of abdominal pain among pregnant women [1]. The effectiveness of ultrasound imaging for diagnosing ureteral stones ranges between 34% and 92% because of difficulty differentiating hydronephrosis caused by ureteral stones from physiologic hydronephrosis, with further effects of a technician's skill [1]. Magnetic resonance imaging (MRI) without contrast provides a suitable alternative for assessing acute abdominal pain in pregnant women. There is no conclusive record of MRI exposure's adverse effects on fetal development [3,4]. Accordingly, MRI without contrast has increasingly been accepted in this clinical context [1].

Herein, we describe a suspected ureteral stone as the cause of acute right lower abdominal pain in a pregnant woman. Ultrasound examination revealed hydronephrosis. However, because of the difficulty in differentiating between ureteral stone and physiologic causes of hydronephrosis, we proceeded with MRI without contrast, which led to a timely diagnosis of idiopathic renal hemorrhage, a rare condition of occult hematuria. Our case demonstrates the usefulness of MRI for diagnosing acute abdominal pain in pregnant women.

## Case presentation

The patient was a 35-year-old woman with a history of 2 miscarriages. At the time of consultation, the patient was in her 10th week of pregnancy through artificial insemination. She was taking 100 mg of aspirin per day. She presented to the emergency department at night with a chief complaint of gross hematuria and right lower abdominal pain, which had developed 2 days prior. Physical examination revealed positive tenderness of the right lower abdomen and costovertebral angle (CVA) tenderness on tapping. Abdominal ultrasound showed hydronephrosis in the right kidney but no visible stone lesions in the pyelonephritic transition zone. Information was provided to the patient and family regarding the high possibility of a ureteral calculus, which could not be confirmed via ultrasound. The physician presented the patient and family with recent evidence on the safety of MRI for pregnant women and the usefulness of MRI for abdominal pain disease, including ureteral calculi, without risk of radiation exposure. Through shared decision-making, we proceeded with MRI without contrast, aligning with the patient's desire for a diagnostic examination.

**Fig. 1:**
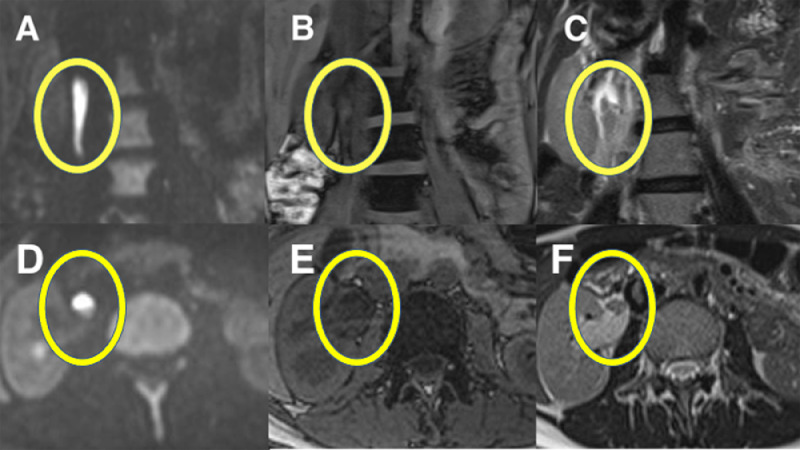
Magnetic resonance imaging shows hydronephrosis of the right kidney and a 4 cm lesion in the renal pelvic-ureteral junction (yellow oval). (A, D) Diffusion-weighted images show high signal intensity, (B, E) T1-weighted images show low signal (with some slightly high-signal intensity) intensity, and (C, F) T2-weighted images show low signal intensity.

MRI showed hydronephrosis of the right kidney and a 4 cm lesion with low signal on T1- and T2-weighted images (half-Fourier acquisition, single-shot turbo spin-echo: HASTE) and high signal on diffusion-weighted images in the lesion in the renal pelvic-ureteral junction (Fig. 1). Symptoms improved with antiemetic and analgesic treatment. An appointment was made for a follow-up with a urologist the following day.

From MRI images and radiology reports and the patient's clinical symptoms, a diagnosis of idiopathic renal hemorrhage and suspected intra-ureteral hematoma was made by the urologist, and the patient was followed up on an outpatient basis. Aspirin was discontinued at the time of the first follow-up. The patient's symptoms improved, with resolution of the occult urine blood within 4 weeks and negative urine cytology. There was no recurrence of hematuria.

## Discussion

In this case, we described our use of MRI for a close abdominal examination of a woman at 10 weeks of pregnancy. MRI was effective for differentiating idiopathic renal hemorrhage, a rare condition, from ureteral stones. Idiopathic renal hemorrhage, also known as "essential hematuria" or "chronic unilateral hematuria," is characterized by intermittent or persistent gross unilateral hematuria from the upper urinary tract [5]. Idiopathic renal hemorrhage is characterized by intermittent or continuous gross hematuria that cannot be diagnosed using standard radiology and laboratory findings. The usefulness of ureteroscopy for differentiating the causes of hematuria, including hemangiomas, microvenous rupture, varicose veins, transitional cell carcinoma, and ureteral calculus, has been recently reported [6–8]. MRI showed a 4 cm occupying lesion with low signal intensity on T1- and T2-weighted images and high signal intensity on diffusion-weighted images in the ureter, which, together with the clinical course, led to the conclusion of an intraurethral acute hematoma due to idiopathic renal hemorrhage. Although we did not perform an endoscopic examination for diagnostic confirmation in our case, we believe our diagnosis of idiopathic renal hemorrhage was acceptable based on the absence of kidney abnormalities on imaging, negative cytology, and the resolution of the hematuria on follow-up. To our knowledge, there are no prior reports of MRI signal changes of intra-ureteral hematomas due to idiopathic renal hemorrhage.

The most frequent cause of idiopathic renal hemorrhage is a hemangioma or microvascular rupture [7,8]. Hematoma formation in the ureter due to hematuria and hydronephrosis secondary to ureteral obstruction may have caused the pain in our patient. The rapid resolution of the hematuria suggests that the idiopathic renal hemorrhage in our case was exacerbated by antiplatelet therapy. MRI without contrast has also shown utility for diagnosing nonobstetric and gynecologic conditions, such as ureteral stones, bowel obstruction, and appendicitis [2]. Therefore, MRI without contrast is recommended as the imaging modality of choice, following ultrasound, for close examination of acute abdominal pain in pregnant women [1]. Although the following risks are associated with MRI in pregnancy, including prolonged scan time, difficulty in image interpretation, and incidentaloma, no maternal or fetal harm has been reported [4]. Therefore, MRI should be considered for acute abdominal pain in pregnant women, and the decision for MRI should be based on sharing information with the patient, the patient's consent, availability, and the clinical situation [9].

In summary, although the use of MRI in diagnosing acute abdominal pain in pregnant women is still not widespread, our case demonstrates the usefulness of MRI for the timely diagnosis of acute abdominal pain, even for the rare condition of idiopathic renal hemorrhage. Further evidence through case reports and research is warranted to validate this clinical use of MRI. Education regarding the appropriate use of MRI will also need to be considered.

## Ethics approval

Our institution does not require ethics approval for reporting individual cases.

## Patient consent

Written informed consent was obtained from the patient for the use of her anonymized clinical information in the publication of this case report.
